# Lid-Driven Chamber with 3D Elliptical Obstacle under the Impacts of the Nano-Properties of the Fluid, Lorentz Force, Thermal Buoyancy, and Space Porosity

**DOI:** 10.3390/nano12142373

**Published:** 2022-07-11

**Authors:** Houssem Laidoudi, Aissa Abderrahmane, Abdulkafi Mohammed Saeed, Kamel Guedri, Obai Younis, Riadh Marzouki, Jae Dong Chung, Nehad Ali Shah

**Affiliations:** 1 Faculty of Mechanical Engineering, University of Sciences and the Technology of Oran, Oran 31000, Algeria; houssem.laidoudi@univ-usto.dz; 2 Laboratoire de Physique Quantique de la Matière et Modélisation Mathématique (LPQ3M), University of Mascara, Mascara 29000, Algeria; a.aissa@univ-mascara.dz; 3 Department of Mathematics, College of Science, Qassim University, P.O. Box 6644, Buraydah 51452, Saudi Arabia; abdulkafi.ahmed@qu.edu.sa; 4 Department of Mathematics, College of Education, Hodeidah University, P.O. Box 3114, Al-Hudaydah 207416, Yemen; 5 Mechanical Engineering Department, College of Engineering and Islamic Architecture, Umm Al-Qura University, P.O. Box 5555, Makkah 21955, Saudi Arabia; kmguedri@uqu.edu.sa; 6 Department of Mechanical Engineering, College of Engineering at Wadi Addwaser, Prince Sattam BinAbdulaziz University, P.O. Box 173, Al-Kharj 11942, Saudi Arabia; oubeytaha@hotmail.com; 7 Chemistry Department, College of Science, King Khalid University, P.O. Box 394, Abha 61413, Saudi Arabia; rmarzouki@kku.edu.sa; 8 Chemistry Department, Faculty of Sciences of Sfax, University of Sfax, Sfax 3038, Tunisia; 9 Department of Mechanical Engineering, Sejong University, Seoul 05006, Korea; nehadali199@sejong.ac.kr

**Keywords:** mixed convection, entropy generation, nanofluid, permeability, MHD

## Abstract

In this work, we have performed an investigation to increase our understanding of the motion of a hybrid nanofluid trapped inside a three-dimensional container. The room also includes a three-dimensional heated obstacle of an elliptic cross-section. The top wall of space is horizontally movable and adiabatic, while the lower part is zigzagged and thermally insulated as well. The lateral walls are cold. The container’s space is completely replete with Al2O3-Cu/water; the concentration of nanoparticles is 4%. The space is also characterized by the permeability, which is given by the value of the Darcy number (limited between 10^−5^ and 10^−2^). This studied system is immersed in a magnetic field with an intensity is defined in terms of Hartmann number (limited between 0 and 100). The thermal buoyancy has a constant impact (Gr = 1000). This study investigates the influences of these parameters and the inclination angle of the obstacle on the heat transfer coefficient and entropy generation. The Galerkin finite element method (GFEM) was the principal technique for obtaining the solution of the main partial equations. Findings from our work may be exploited to depict the conditions for which the system is effective in thermal cooling and the case in which the system is effective in thermal insulation.

## 1. Introduction

A heated baffle inside a liquid-filled chamber is a popular field of investigation. It is used in either cooling systems for thermal transformers and heating devices or in thermal insulation systems. Because of the importance of these systems in daily life, researchers have provided interesting results from these systems under various initial conditions [1,2,3,4,5,6,7,8,9,10].

Most of these studies focus on the study of an open room [11,12,13], with a wavy shape then inserted between the room’s walls [14,15,16,17]. In order to improve the thermal transfer process between the inner obstacle and the flow, different shapes have been tested, such as triangular [18,19], square [20,21] and hexagonal [22] forms.

Several recent studies have used nanofluids as a new technique to improve the thermal transfer process [23,24,25,26]. This type of fluid depends mainly on adding very fine solid particles of metals to raise the fluid’s thermal proprieties without changing its hydrodynamic behavior.

Chamkha et al. [27] investigated a confined nanofluid in a cone-shaped room. In the middle of this container, a circular obstacle regularly rotates. The interior of the room is porous. This system was also subjected to a magnetic field. Among the most important criteria that have proven to be effective in increasing heat transfer is the blockage ratio, which was found to be able to raise the heat transfer rate by up to 95%. Selimefendigil and Chamkha [28] studied the hybrid nanofluid (Ag–MgO/H_2_O) in a square container under the presence of thermal buoyancy, magnetohydrodynamics, and space porosity. The results of this work have proven that these parameters have a clear effect on the fluid movement inside the container and thus directly affect the thermal transfer. Sun et al. [29] numerically examined double diffusion using triangular fins of conductive performance in a lid-driven room. They proved that the triangular fin is a suitable control criterion for the flow structure and heat transfer rate. Elatar et al. [30] generated the heat by using natural convection in a laminar regime in a square room with an adiabatic horizontal part with a single horizontal fin at different lengths and places connected to the heated source. They tested the impact of fin length and frame placement on flow trajectory and heat patterns. Laidoudi and Helmaoui [31] studied the natural convection from an elliptical obstacle located in a circular chamber. The most important point considered here is the positioning of the inner obstacle. It has been verified that the angle of inclination of this type of obstacle greatly affects the thermal transfer.

In addition, there are other similar works to these studies that aim to determine the fluid’s heat transfer activity [32,33,34,35]. Lv et al. [36,37,38,39] have completed a series of investigations on the cooling process inside a micromixer. 

Through the foregoing studies, it has been found that:

- The use of solid metal particles in a liquid enhances its ability to conduct heat.
- The application of a magnetic field on a flow affects its trajectory and velocity, which either negatively or positively effects the thermal transfer rate.
- Using zigzagged walls for rooms instead of flat walls enhances heat transfer.
- Using an elliptical shape for the baffles instead of a circular shape may improve heat transfer.
- This study is a 3D heated elliptical obstacle enclosed in the middle of a chamber. The lower end of the room is zigzagged, while the upper end is horizontally movable. The space between the room walls and the elliptical obstacle is filled with a hybrid nanofluid containing 4% nanoparticles (Al_2_O_3_-Cu/water). The magnetic field is also applied to the present system.

Moreover, the space is also considered to be porous. The contours of isotherm, entropy generation, and pathlines are depicted to explain the thermal and dynamic patterns of the flow. In addition, the average Nu and Be numbers are plotted versus all studied parameters.

## 2. Mathematical Model and the Study Configuration

**Figure 1:**
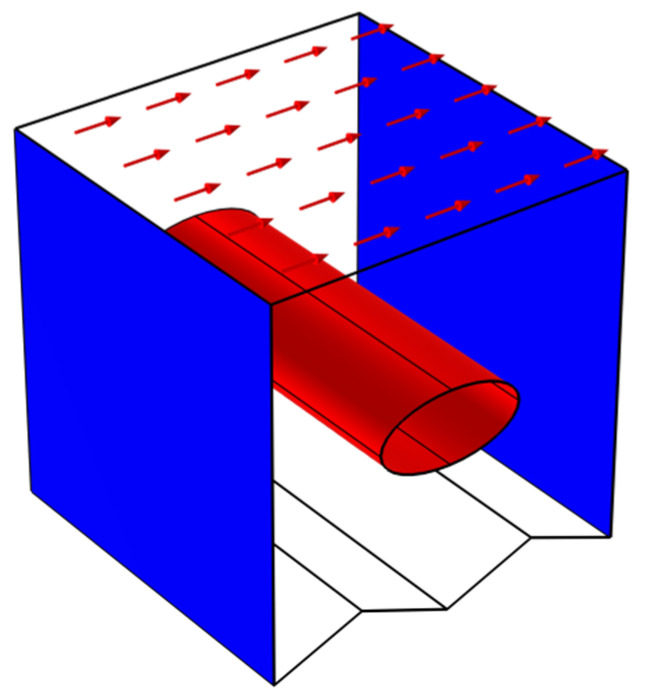
The physical domain and the boundary conditions.

In Table 1, the values of thermophysical characteristics of nanofluid elements are presented. In Figure 1, the proposed configuration is depicted as a 3D-zigzagged porous cavity containing a nanofluid with a magnetic force applied along the positive y- and z-axes. Except for the zigzagged wall, which is considered at the hot temperature designated (Th), and the front wall, which is considered at the cold temperature denoted (C), all walls are assumed to be adiabatic (Tc). The rough wall is assumed to be the primary geometry influencer and will have multiple undulations (N = 4, 2, and 1). The upper wall travels at a constant speed, U, in the opposite direction.

### 2.1. Mathematical Model

The mathematical model refers to flow inside a 3D porous cavity, and the selected liquid is a Newtonian-incompressible fluid undergoing a laminar regime [35]:

The conservation equations are:

(1) $$\frac{\partial U}{\partial X}+\frac{\partial V}{\partial Y}+\frac{\partial W}{\partial Z}=0$$

The momentum equations along the three directions are:

(2) $$\begin{array}{l} \frac{\rho_{nf}}{\rho_{f}}\left[\frac{U}{\varepsilon^{2}}\frac{\partial U}{\partial X}+\frac{V}{\varepsilon^{2}}\frac{\partial U}{\partial Y}+\frac{W}{\varepsilon^{2}}\frac{\partial U}{\partial Z}\right]\\ \;\;\;\;\;=-\frac{\rho_{nf}}{\rho_{f}}\frac{\partial P}{\partial X}+\frac{1}{Re}\frac{1}{\varepsilon }\frac{\mu_{nf}}{\mu_{f}}\left(\frac{\partial U}{\partial X}+\frac{\partial U}{\partial Y}+\frac{\partial U}{\partial Z}\right)-\frac{\mu_{nf}}{\mu_{f}ReDa}U-\frac{\rho_{nf}}{\rho_{f}}\frac{0.55}{\sqrt{Da}}\sqrt{U^{2}+V^{2}+W^{2}}U \end{array}$$

(3) $$\begin{array}{l} \frac{\rho_{nf}}{\rho_{f}}\left[\frac{U}{\varepsilon^{2}}\frac{\partial V}{\partial X}+\frac{V}{\varepsilon^{2}}\frac{\partial V}{\partial Y}+\frac{W}{\varepsilon^{2}}\frac{\partial V}{\partial Z}\right]=-\frac{\rho_{nf}}{\rho_{f}}\frac{\partial P}{\partial Y}+\frac{1}{Re}\frac{1}{\varepsilon }\frac{\mu_{nf}}{\mu_{f}}\left(\frac{\partial V}{\partial X}+\frac{\partial V}{\partial Y}+\frac{\partial V}{\partial Z}\right)\\ -\frac{\mu_{nf}}{\mu_{f}ReDa}V-\frac{\rho_{nf}}{\rho_{f}}\frac{0.55}{\sqrt{Da}}\sqrt{U^{2}+V^{2}+W^{2}}V-\frac{\sigma_{nf}}{\sigma_{f}}Ha^{2}\frac{V}{\varepsilon } \end{array}$$

(4) $$\begin{array}{l} \frac{\rho_{nf}}{\rho_{f}}\left[\frac{U}{\varepsilon^{2}}\frac{\partial W}{\partial X}+\frac{V}{\varepsilon^{2}}\frac{\partial W}{\partial Y}+\frac{W}{\varepsilon^{2}}\frac{\partial W}{\partial Z}\right]=-\frac{\rho_{nf}}{\rho_{f}}\frac{\partial P}{\partial Z}+\frac{1}{Re}\frac{1}{\varepsilon }\frac{\mu_{nf}}{\mu_{f}}\left(\frac{\partial W}{\partial X}+\frac{\partial W}{\partial Y}+\frac{\partial W}{\partial Z}\right)\\ -\frac{\mu_{nf}}{\mu_{f}ReDa}W-\frac{\rho_{nf}}{\rho_{f}}\frac{0.55}{\sqrt{Da}}\sqrt{U^{2}+V^{2}+W^{2}}W+\frac{{\left(\rho \beta \right)}_{nf}}{{\left(\rho \beta \right)}_{f}}Ri\theta -\frac{\sigma_{nf}}{\sigma_{f}}Ha^{2}\frac{W}{\varepsilon } \end{array}$$

where the last terms in Equations (3) and (4) are the Lorentz force. The heat equation is:

(5) U∂θ∂X+V∂θ∂X+W∂θ∂Z=(ρcP)f(ρcP)nfkeffkf1RePr[∂2θ∂X2+∂2θ∂Y2+∂2θ∂Z2]

where $k_{eff}=(1-\varepsilon )k_{s}+\varepsilon k_{nf}$ (*k*_s_ refers to the solid thermal conductivity for the matrix of the porous layer, *k*_s_ = 0.78 W/m·K, and ε = 0.37)

$$X,Y,Z=\frac{x,y,z}{L},\;U,V,W=\frac{(u,v,w)L}{\alpha_{nf}},\;\theta =\frac{T-T_{c}}{T_{h}-T_{c}},$$

$$P=\frac{pL^{2}}{\rho_{nf}\alpha_{fl}^{2}},Pr=\frac{v_{f}}{\alpha_{f}},\;Da=\frac{K}{L^{2}}$$

$$Ra=\frac{g\beta_{f}(T_{h}-T_{c})L^{3}}{\alpha_{f}v_{f}},\;Ha=LB\sqrt{\frac{\sigma_{nf}}{\mu_{nf}}},$$

where ε is the porosity,

$$Ri=\frac{Gr}{Re^{2}}$$

**Table 2:** The physical meaning of the dimensionless numbers.

**Dimensionless Number**	**Physical Meaning**
Reynolds (Re)	Determines wall speed
Grashof (Gr)	Determines the thermal buoyancy strength
Richardson (Ri)	Determines the ratio between natural and forced convection
Darcy (Da)	Shows the permeability of the space
Hartmann (Ha)	Controls the strength of the magnetic field

The hybrid nano-liquid thermophysical properties are shown in Table 3.

### 2.2. Boundary Conditions

#### Total Entropy Generation

The dimensionless form of the total entropy generation $S_{tot}$ is expressed as follows [37]:

(6) $$S_{TOT}=S_{HT}+S_{FF}+S_{MF}$$

where:

(7) $$S_{HT}=\frac{k_{hnf}}{k_{fluid}}\left[{\left(\frac{\partial \theta }{\partial X}\right)}^{2}+{\left(\frac{\partial \theta }{\partial Y}\right)}^{2}+{\left(\frac{\partial \theta }{\partial Z}\right)}^{2}\right]$$

(8) $$\begin{array}{l} S_{FF}=\frac{\mu_{hnf}}{\mu_{fluid}}\varphi \left[\begin{array}{l} 2{\left(\frac{\partial U}{\partial X}\right)}^{2}+2{\left(\frac{\partial V}{\partial Y}\right)}^{2}+2{\left(\frac{\partial W}{\partial Z}\right)}^{2}\\ +{\left(\frac{\partial U}{\partial Y}+\frac{\partial V}{\partial X}\right)}^{2}+{\left(\frac{\partial W}{\partial Y}+\frac{\partial V}{\partial Z}\right)}^{2}\\ +{\left(\frac{\partial U}{\partial Z}+\frac{\partial W}{\partial X}\right)}^{2}+\frac{U^{2}+V^{2}+W^{2}}{Da} \end{array}\right] \end{array}$$

and

(9) $$S_{MF}=\varphi \frac{\sigma_{hnf}}{\sigma_{fluid}}\frac{Ha^{2}}{\varepsilon }\left(W^{2}+V^{2}\right)$$

where $\varphi =\frac{\varepsilon \mu_{nf}T_{0}}{k_{eff}}{\left(\frac{\alpha_{nf}}{L\Delta T}\right)}^{2}$, with $T_{0}=\frac{T_{h}+T_{c}}{2}=0.5$ and $\Delta T=T_{h}-T_{c}$.

The dimensionless form of the Bejan number is as follows:

(10) $$Be=\frac{S_{HT}}{S_{TOT}}$$

The local and average Nusslet numbers are calculated as follows:

(11) $$Nu_{loc}=-\frac{k_{eff}}{k_{fl}}\frac{\partial \theta }{\partial S};\;Nu_{avg}=\frac{1}{S}\int_{0}^{S}Nu\;dxdz$$

## 3. Numerical Method and Validation

### 3.1. Computation Procedure

The main partial Equations (1)–(3) were numerically solved using the appropriate boundary conditions(Table 2) to derive each of the Equations (4)–(10) (Table 4). The Galerkin-weighted residual finite element approach [38] was used to solve the equations. Mesh dependency was investigated using a variety of grids. A grid of 629215 elements was used for the current simulations (Table 5).

The finite element method based on the Newton technique was used to discretize and solve the governing equations in a grid composed of triangular elements. The convergence of the solution is only adequate if the following convergence criterion for the relative error of each variable is achieved:

(12) $$\left|\frac{\Gamma^{i+1}-\Gamma^{i}}{\Gamma^{i+1}}\right|\leq \eta$$

where i indicates the iteration value and η represents the convergence criterion. In this numerical study, the convergence criterion was defined as $\eta =10^{-6}$.

### 3.2. Validation

**Figure 2:**
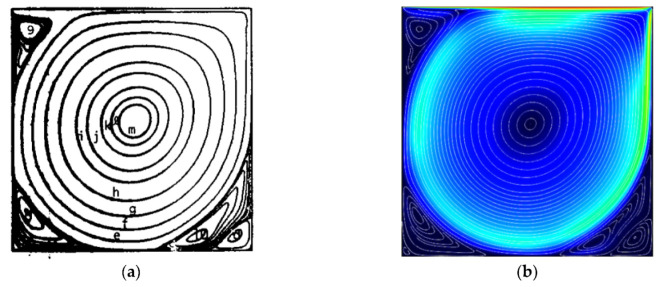
Streamlines (Ghia et al. [39] (**a**)) and Present study (**b**).

To ensure that the numerical technique used to convert the authorized code is correct, the velocity profile inside the enclosure with obstructions is compared to the work of Ghia et al. [39], Figure 2:

## 4. Results and Discussion

The results of this research aim to expand the understanding of the heat transfer process between an elliptical cylinder’s external surface and a nanofluid. This thoughtful system is confined in a room. The upper wall moves horizontally at a constant speed and under adiabatic conditions, while the rest of the walls are stationary. In addition, the lower wall of the container is zigzagged and thermally insulated (adiabatic surface). The value of the Reynolds number (Re) is a determinant of the speed of the movement of the upper wall. In fact, the horizontal movement of the wall serves to move the fluid within the chamber.

For the heat transfer mechanism, the horizontal walls of the room are cold, while the cylinder wall is hot. That is, the studied fluid (hybrid nanofluid) in this system acts as a thermal medium, transferring thermal energy between the cylinder and the cold horizontal walls. Moreover, the interior medium of the container is characterized by fluid permeability, the value of which is expressed in the Darcy (Da) number. That is, the greater this value, the better the permittivity of the medium to cross the fluid flow. In addition, this system is placed inside an external magnetic field where a new force called the Lorentz force is generated. The intensity of this force is controlled by the Hartmann (Ha) number.

Richardson’s number (Re) is defined by the following expression, Ri = Gr/(Re^2^), where Gr = 1000. This means that the heat transfer combines natural and forced convection. The natural convection is superior to the forced convection for Re = 1 and 10, whereas the forced convection predominates for Re = 100 and 500. 

We should mention that the main points studied in this work are: the value of Da (=10^−5^, 10^−4^, 10^−3^, 10^−2^), which determines the permeability of the space; the value of Re (=1, 10, 100, and 500) defined for the speed movement of the upper wall; the angle of inclination of the elliptical obstacle (=0, 30, 60, and 90); and the value of Ha (=0, 25, 50, 75, and 100), which indicates the intensity of the Lorentz force. The nanoparticles used here are 4% Al_2_O_3_-Cu/water.

**Figure 3:**
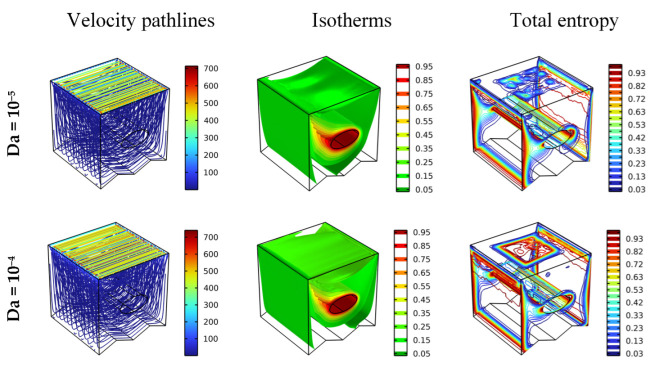

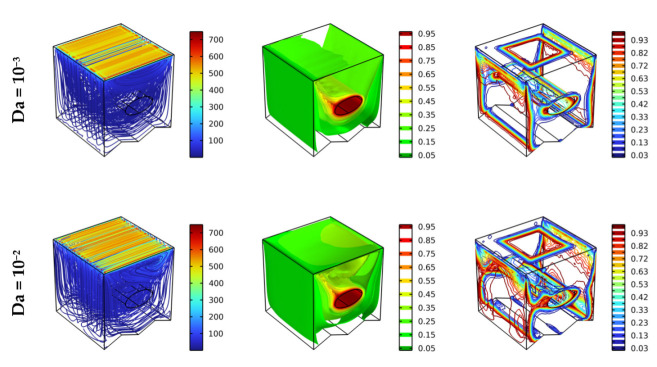
Distribution of the velocity pathlines, isotherms, and total entropy for different Da in different scenarios for Ha = 0, φ = 0.04, and Re = 100.

Figure 3 depicts the impact of Da number (=10^−5^, 10^−4^, 10^−3^, 10^−2^) on the characteristic contours of the pathline, the generation of total entropy, and the isotherm for Ha = 0, γ = 0, and Re = 100. In fact, Figure 3 shows how the permeability of the annular space affects the movement of the nanofluid particles, the thermal distribution, as well as the total entropy generation. Since the movement of the upper wall is from left to right, there is a circular flow movement in the clockwise direction. The fluid velocity is low near the lower wall and increases gradually as we move towards the moving upper wall. It is also observed that the movement of the nanofluid particles increases as the value of Da increases because the annular space becomes more permeable to the fluid. This is confirmed by the gradient temperature around the elliptical obstacle, which gradually increases in terms of the Da number. So, we conclude that by increasing the Da number, the heat transfer of the inner obstacle increases. In addition, the presence of thermal distribution in the form of the plume on the left side indicates that the heat transfer from the right side of the cylinder is better than on the left side. As for the total entropy generation, its basic distribution is observed near the top wall due to the movement of fluid particles and near the cylinder and lateral walls due to the presence of thermal activity. Generally, the total entropy generation in these zones is seen to be augmented with increasing Da. We can conclude that the faster the flow, the greater the transfer of the heat energy; thus, the thermal activity becomes more effective.

**Figure 4:**
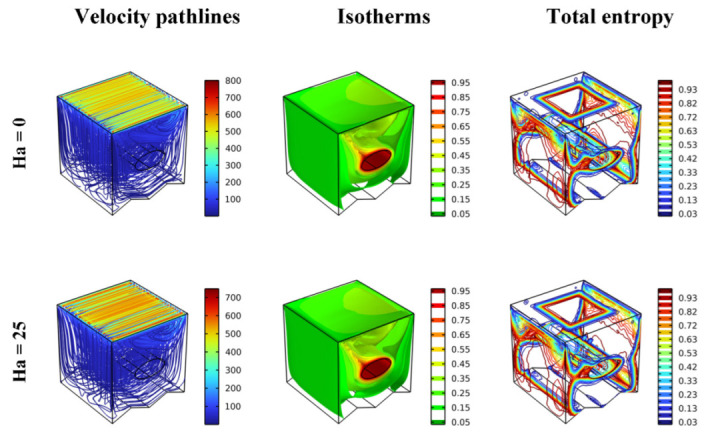

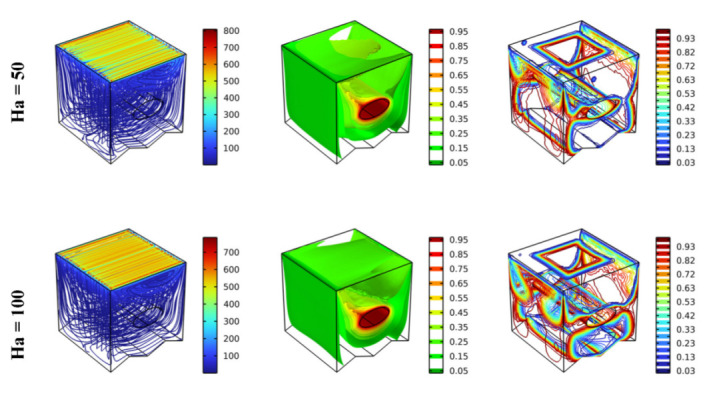
Distribution of the velocity pathlines, isotherm, and total entropy for different Ha in different scenarios for Da = 10^−2^, φ = 0.04, and Re = 100.

The gradual effect of the Hartmann number (=0, 25, 50, 75, and 100) on each of the streamline contours, isotherms, and total entropy generation for Re = 100, γ = 0, and Da = 10^−2^ are well presented in Figure 4. Overall, there is no significant effect of the values of Ha number on the displayed items. Therefore, it can be concluded that changes in this number (Ha) do not cause sensitive changes in the value of the Nusselt number. The value of the Ha number indicates the existence of a force (Lorentz force) applied mainly in the opposite direction of the flow, which somewhat hinders the movement of fluid particles and results in a decrease in the thermal transfer rate. The Lorentz force affects the opposite of the movement of the flow, which means a decrease in the flow’s speed, making the heat transfer more difficult.

**Figure 5:**
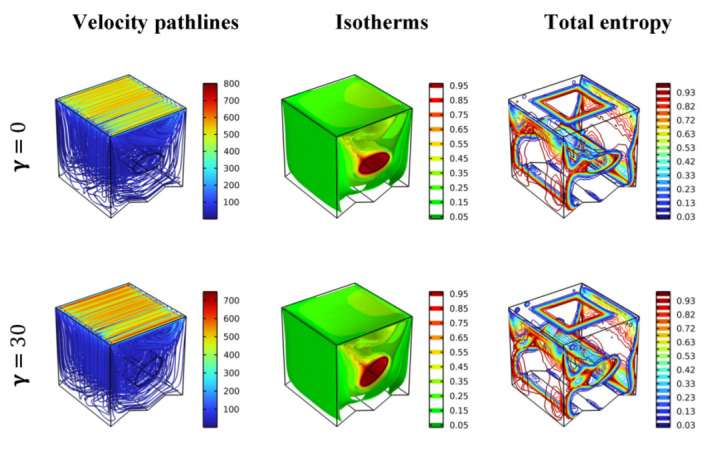

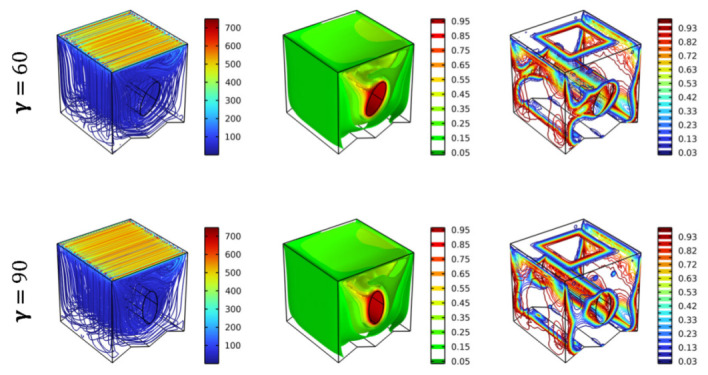
Distribution of the velocity pathlines, isotherm, and total entropy in different scenarios for Ha = 0, Da = 10^−2^, φ = 0.04, and Re = 100.

Figure 5 presents the impact of the inclination angle (=0, 30, 60, 90) of the elliptical cylinder on the streamlines, dimensionless temperature (isotherms), and the contours of total entropy generation for Re = 100, Ha = 0, and Da = 10^−2^. From the streamlines, the displacement of the elliptical obstacle from 0 to 90 degrees makes it more streamlined, making the streamlines more stable and balanced. This observation is reinforced by the increase in the gradient temperature around the cylinder as the angle of the inclination increases. This indicates that the heat transfer, in this case, increases with increases in the angle of inclination. Since the cylinder’s rotation allows the nanofluid’s movement and enhances the thermal activity, it is also noted that the total entropy generation near the cylinder and the walls increase with the increase in obstacle inclination.

**Figure 6:**
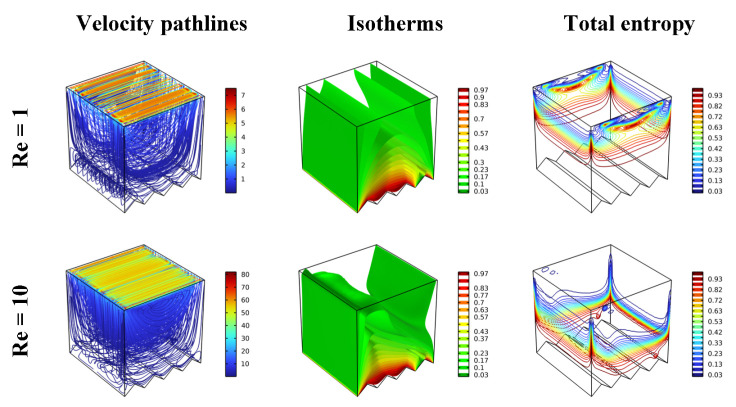

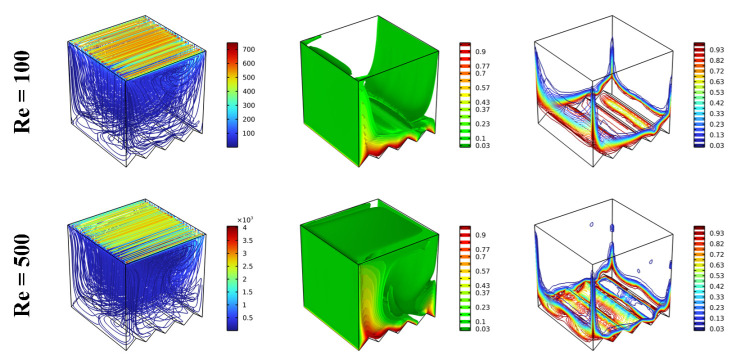
Distribution of velocity pathlines, isotherm, and total entropy for different Re in different scenarios for Ha = 0, Da = 10^−2^, and φ = 0.04.

The influence of the Re number value (=1, 10, 100, and 500) on the previously presented elements (contours) for Da = 10^−2^ and Ha = 0 is depicted in Figure 6. Clearly, the higher value of the Re number leads to an increase in the velocity of the upper wall and interference in the fluid’s viscosity. The fluid layers move each other, creating a recirculation flow. Through the isotherms, it is noted that the increase in the flow velocity makes the distribution of gradient temperature around the obstacle more important, and accordingly, the rate of thermal transfer increases gradually in terms of Re. As for the positional distribution of the total entropy generation, it is seen that the higher the value of the Re number, the higher the total entropy around the inner obstacle and also near the sides of the horizontal walls and the upper wall.

**Figure 7:**
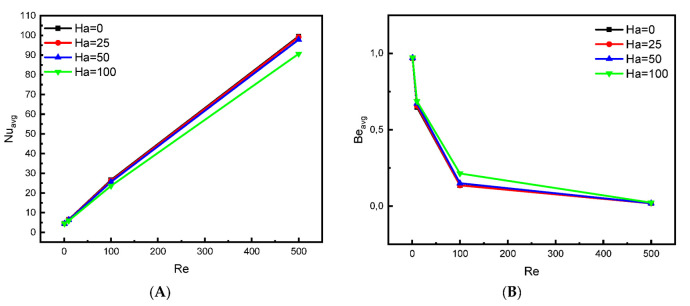
Effect of Ha on (**A**) Nu_avg_ and (**B**) Be_avg_ for Da = 10^−2^, ϕ=0.04.

Figure 7A presents the development of the mean Nu number for the elliptical obstacle in terms of Re (=1, 10, 100, and 500) and Ha (=0, 25, 50, and 100) for Da = 10^−2^ and inclination angle (γ = 0). As expected from the analysis of the previous results, the increase in the speed of the movement of the upper wall increases the velocity of the flow, which results in the acceleration of thermal transfer. Therefore, we find that the value of Nu value increases analogously with the value of Re. On the other hand, Lorentz force intensity acts against the movement of the nanofluid flow, which causes a decrease in the velocity and a consequent reduction in the thermal transfer process, and this is explained by the development in the values of Nu number in terms of Ha. Figure 7B presents the mean value of the Be number in terms of Ha and Re. For the low values of Re (1 and 10), the entropy produced by the temperature is greater than that by the dynamic movement of the flow source. Whereas for the values of 100 and 500 for Re, the dynamic source becomes dominant.

**Figure 8:**
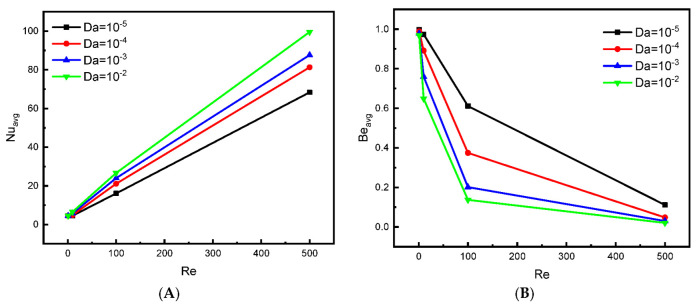
Effect of Da on (**A**) Nu_avg_ and (**B**) Be_avg_ for ϕ=0.04, Ha = 0.

Figure 8A presents the effect of enclosure permeability on the value of the obstacle’s Nu number for all studied values of Re number. These results confirm our previous analysis: the higher the permeability of the annular space (increasing Da), the higher the flow speed, which positively reflects the speed of thermal evacuation. Therefore, Figure 8A shows that the value of Nu increases proportionally as the value of Da or Re increases. On the other hand, Figure 8B describes the change in Be number in terms of Re and Da. It is noticed that whenever the permeability of the space is low, the value of Be number is significant. This can be explained by the following: when the fluid particles move less, most of the entropy generation produced is due to the heat source.

**Figure 9:**
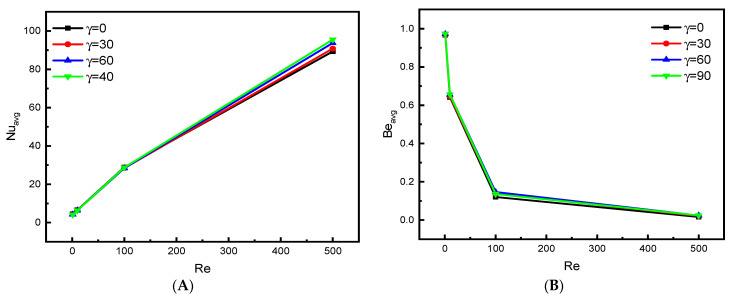
Effect of N on (**A**) Nu_avg_ and (**B**) Be_avg_ for ϕ=0.04, Ha = 0, Da = 10^−2^.

Figure 9A presents the effect of the inclination angle of the elliptical obstacle on the values of Nu of the obstacle itself for Ha = 0 and Da = 10^−2^. It is evident from this Figure 9A that the angle of inclination has an effect on the value of the Nu number. That is, the greater the inclination of the obstacle, the greater the value of Nu. From here, it can be concluded that an angle of zero (γ = 0) for the elliptical obstacle is very effective in industrial heat-insulating applications, while an angle of ninety (γ = 90) is effective in the cooling system. Figure 9B shows the variation in Be versus the inclination angle (γ = 0, 30, 60, 90) and Re. There is an approximate correspondence between the values of Be in terms of the inclination angle of the obstacle.

## 5. Conclusions

The present work employs a simulation model of a confined nanofluid flowing around a hot elliptical obstacle. The flow conditions are: the annular space is porous; the bottom wall is zigzagged; the upper part of the container is moving horizontally; the lateral walls have a low temperature; a magnetic field is considered to be applied to the system; and finally, the elliptical obstacle is subjected to an angle of inclination. The results focused on: the angle of inclination of the obstacle (γ = 0 to 90); the porosity of the annular space (Da = 10^−5^ to 10^−2^); the intensity of the magnetic field (Ha = 0 to 100) and the speed movement of the upper part (Re = 1 to 100). We highlight the appropriate conditions for increasing the thermal activity for the applications related to refrigeration and identify the suitable conditions for insulating applications. Overall, we confirmed the following points:

- Increasing the value of Da and/or Re increases the average Nu of the obstacle, and therefore the heat transfer rate increases in terms of these elements. Here we can say that increasing the speed of wall movement and/or the porosity of the space is suitable for cooling activities.
- The magnetic field applied to the present system decreases the Nu value of the elliptical obstacle and hampers the thermal transfer activity. Hence, this case can be applied to thermal insulators.
- Rotating the cylinder from the horizontal position to the vertical position increases the heat transfer; therefore, the first case is suitable for thermal insulation cases, while the second is the best for cooling applications.

A base fluid of the non-Newtonian type can be proposed for future work.

## Figures and Tables

**Table 1:** Thermophysical properties of ${\mathrm{Al}}_{2}O_{3},\;\mathrm{Cu}$, and water.

Thermophysical Properties	${\mathrm{Al}}_{2}O_{3}$	Cu	water
$\rho \left(kg/m^{3}\right)$	3970	8933	997.1
$C_{p}\left(J/kgK\right)$	765	385	4179
k(W/mK)	40	400	0.613
σ(S/m)	$3.69\times 10^{7}$	$5.96\times 10^{7}$	0.05

**Table 3:** Correlations that define the hybrid nano-liquid thermophysical properties [36].

Property	Correlation
Density	$\rho_{nf}=\left(1-Ø\right)\rho_{f}+Ø\rho_{np}$
Heat capacity	$c_{p}{}_{nf}=\left(1-Ø\right)c_{p}{}_{f}+Øc_{p}{}_{np}$
Coefficient of thermal dilatation	$\beta_{nf}=\left(1-Ø\right)\beta_{f}+Ø\beta_{np}$
Electrical conductivity	$\sigma_{nf}=\left(1-Ø\right)\sigma_{f}+Ø\sigma_{np}$
Thermal conductivity	$k_{nf}=\frac{k_{np}+\left(n-1\right)k_{f}-\left(n-1\right)\left(k_{f}-k_{np}\right)Ø}{k_{np}+\left(n-1\right)k_{f}+\left(k_{f}-k_{np}\right)Ø}k_{f}$
Viscosity	$\mu_{nf}=\frac{\mu_{f}}{{\left(1-Ø\right)}^{2.5}}$

**Table 4:** Boundary conditions for our study.

	Thermal Condition	Velocity Condition
The left wall	θ=0	U,V,W=0
The right wall	θ=0	U,V,W=0
The top and bottom wall	adiabatic	U=1,V,W=0
The top wall	adiabatic	U,V,W=0
The inner cylinder wall	θ=1	U,V,W=0

**Table 5:** Nu_avg_ and Be_avg_ for different mesh sizes for Re = 10.

No. of Grid Elements	53,274	93,700	218,558	629,215	2,576,359
Nu_avg_	6.6421	6.5563	6.5515	**6.5432**	6.5429
Be_avg_	0.64964	0.64951	0.64832	**0.64832**	0.64831

## Data Availability

The data used to support the finding of this study are included within the article.
